# A Rare Case of Adductor Longus Muscle Rupture

**DOI:** 10.1155/2015/840540

**Published:** 2015-03-30

**Authors:** R. J. L. L. van de Kimmenade, C. J. A. van Bergen, P. J. E. van Deurzen, R. A. W. Verhagen

**Affiliations:** ^1^Department of Orthopaedics & Traumatology, Tergooi Hospital, Riebeeckweg 212, 1213 XZ Hilversum, Netherlands; ^2^Department of Radiology, Tergooi Hospital, Riebeeckweg 212, 1213 XZ Hilversum, Netherlands

## Abstract

An adductor longus muscle rupture is a rare injury. This case report describes a 32-year-old patient with an adductor longus rupture. The trauma mechanism was a hyperabduction movement during a soccer game. Nonoperative treatment was initiated. After a follow-up of 4 years, the patient was without pain but a small swelling was still visible. This report describes the anatomy, pathophysiology, and evidence-based treatment of adductor longus rupture.

## 1. Introduction

**Figure 1:**
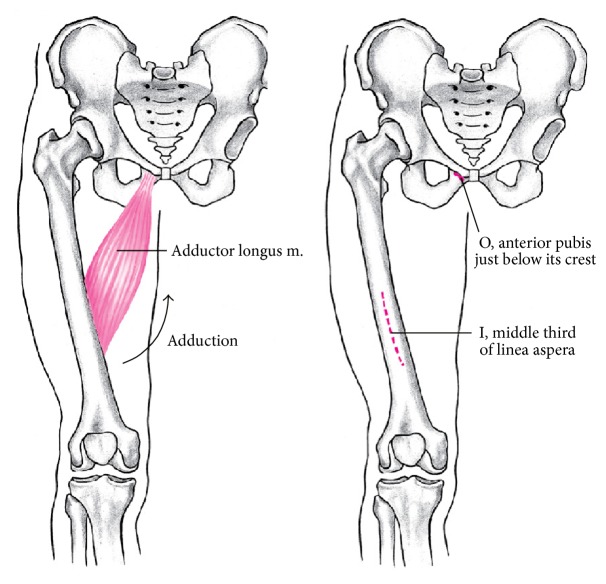
Anatomy of the adductor longus muscle.

The adductor longus muscle is a triangular shaped long and relatively thin muscle. The muscle arises from the ramus superior of the pubic bone and is inserted onto the middle part of the linea aspera. The muscle fibres are narrow proximally and straggle more distally (Figure 1). The anterior branch of the obturator nerve innervates the muscle. It is involved in adduction, external rotation, and anteversion of the hip joint.

Subtotal or total ruptures of the adductor longus muscle are rare [1–7]. This injury should be included in the differential diagnosis in patients with chronic groin pain or previous injuries. An important finding is a history of trauma before the onset of pain in the groin region. A common physical finding is the presence of a swelling on the inner side of the thigh.

Due to the low incidence of this injury reports on the treatment are scarce in the literature [1–7]. We pursued to describe an uncommon case and summarize the available literature.

## 2. Case Report

A 32-year-old man presented in our outpatient clinic with pain in the right groin and proximal thigh, which had been present for three months. The symptoms had started during a soccer game. After kicking the ball with his right foot, he felt an acute pain in the groin and proximal thigh. At the same time he experienced some sort of “popping/cracking sensation” in the upper thigh. Following the injury, he was unable to continue playing. He had no history of groin pain or adductor problems before this injury. Intensive physiotherapy after the incident gave no relief of his complaints. When the patient presented to our clinic he still complained of pain and weakness of the right leg.

At the physical examination there was no swelling or hematoma. Range of motion of both hip joints was normal and without pain. During contraction of the adductors against resistance there was a palpable mass at the inner proximal thigh with a dimple being more distal. Testing of the hamstrings showed no abnormalities.

**Figure 2:**
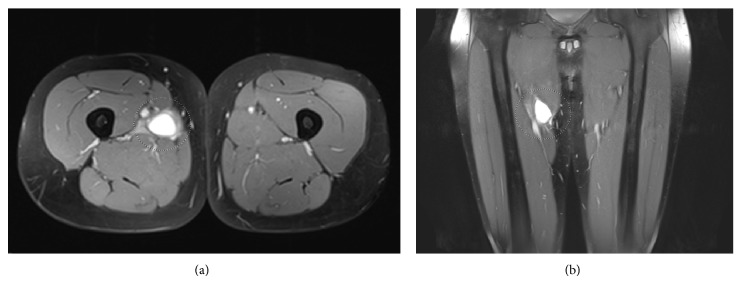
(a) T2-weighted transversal MRI image shows a hyperintense area in the right m. adductor longus; this is characteristic in ruptures of a muscle. (b) T2-weighted coronal MRI image, additional to (a).

Conventional radiographs of the pelvis and the right hip showed no osseous abnormalities. Magnetic resonance imaging (MRI) revealed an intramuscular hyperintense region in the right adductor longus, with a craniocaudal length of 4.2 centimetres. There was no retraction of the muscle. On the basis of the clinical findings and MRI examination the diagnosis was made: an intramuscular adductor longus rupture (Figure 2).

Conservative treatment was started, consisting of a physiotherapy focused on stretching of the muscle followed by strength training. After eight weeks, the complaints had resolved and the patient had resumed playing soccer.

Four years later, the patient presented with a persistent swelling on the inner side of his thigh without pain.

**Figure 3:**
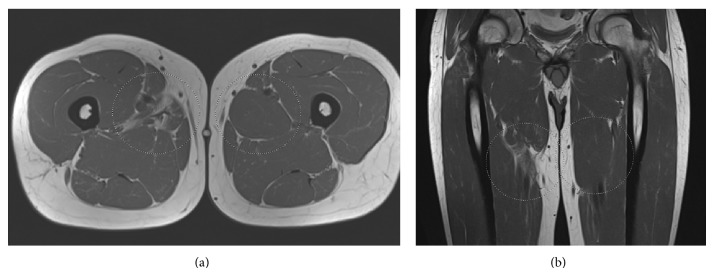
(a) T1-weighted transversal MRI image shows a minimal hyperintense area where the rupture in the adductor longus was seen on previous MRI images after the initial trauma. (b) T1-weighted coronal MRI image, additional to (a).

Additional MRI examination showed subtle higher signal on T1-weighted images in the previous adductor longus rupture, without any structural abnormalities (Figure 3). The findings were discussed with the patient and no further treatment was initiated.

## 3. Discussion

In this paper we describe an uncommon case of a patient with a rupture of the adductor longus muscle, which is a rare injury [2, 3].

One should consider an adductor longus tear in patients with pain in the groin region [1, 3–5]. An important finding of rupture is the presence of a trauma prior to the onset of complaints. The trauma mechanism can give a clue to the diagnosis. A typical trauma mechanism is an eccentric overload caused by forced abduction during contraction of the adductor muscle group [2]. Combinations with extension and endorotation have also been described [3]. Besides the pain, a hematoma and weakness of the adductors can be present. In cases with a tumour-like swelling in the proximal medial part of the thigh, an old rupture of the adductor longus muscle should be considered [1]. An important differential diagnosis is a strangulated inguinal hernia. This usually causes acute pain in the groin region [6]. An important difference in such case is the absence of a trauma.

Little is known about the treatment of an isolated rupture of the adductor longus. Schlegel et al. treated 19 national football league patients, of whom 14 underwent conservative treatment and 5 underwent operative one [4]. All players had a rupture of the adductor longus on the proximal side. Twelve (63%) had experienced some form of antecedent symptoms or event. The nonoperative treatment protocol differed between patients. All operative repairs were performed acutely using suture anchors. The postoperative rehabilitation protocol included protected weight bearing for 2 to 4 weeks and physiotherapy for gaining muscle strength after 6 to 8 weeks. Mean time to return to play was 6.1 weeks for the nonoperative group and 12.0 weeks for the operative group (*P* = 0.001). One player in the operative group had a wound infections and symptomatic heterotopic ossifications.

Sangwan et al. described an 18-year-old man with a mildly painful swelling following a trauma six months earlier [3]. Surgical repair of the ruptured ends was not possible due to retraction. The ruptured proximal muscle was excised and the distal mass was attached to the underlying adductor magnus muscle. Postoperatively there were no complications or disabilities after a follow-up of 18 months.

Vogt et al. described a 43-year-old healthy triathlete with an abduction trauma during skiing [2]. MRI examination revealed a complete osseous avulsion of the adductor longus muscle from the insertion on the pubic bone. The osseous fragment was retracted 3 centimetres from the symphysis. Using three titanium corkscrews, the osseous fragment was reattached. Postoperative treatment existed in a hip orthosis with the hip in 45° flexion for six weeks. The patient returned to full activity without pain in 8 weeks. Physical examination after 3, 6, and 24 months showed a range of motions equal to the contralateral side, no tenderness over the adductor muscle group, and normal strength.

Different studies analyzed the contribution and the function of different muscle groups of the lower extremity in sprinting and cutting manoeuvres. A study by Mann et al. showed that the adductor longus had minimal activity during sprinting [8]. During cutting maneuvers the adductor group is there for stabilization rather than providing power for motion [9]. This seems to indicate that securing and an anatomical reconstruction of the adductor longus may lead to a better outcome.

In other intramuscular injuries, for instance, gastrocnemius tears, conservative treatment is the first choice of treatment [10]. Healing occurs anywhere from 3–6 weeks with comprehensive rehabilitation. Surgical interventions are preserved in patients complicated with the onset of myositis ossificans or in the acute phase associated with an acute compartment syndrome [10].

Tendon ruptures of the gastrocnemius muscle are a common injury of the lower extremity. Treatment of an acute Achilles tendon rupture can be conservative and operative. Soroceanu et al. [11] performed a meta-analysis to compare surgical and conservative treatment of this specific injury. Besides rerupture rates, they also looked at overall rate of other complications and functional outcome. The meta-analysis demonstrated that conservative treatment should be considered at centers using functional rehabilitation. This resulted in rerupture rates similar to those for surgical treatment while offering the advantage of a decrease in other complications. The functional outcomes were similar in both groups; there were no significant differences.

In the present case of an intramuscular rupture, conservative treatment showed good clinical result. There was no pain but a small swelling was still visible after 4 years. Because of lack of evidence in the literature, treatment of this rare injury is individualized. Patient characteristics (age, sport-activities) should be taken into account, as well as the localisation of the rupture (intramuscular or tendineus). More studies are warranted to find out the optimal treatment for ruptures of the adductor longus muscle.
